# Effects of *Abelmoschus manihot* (L.) and its combination with irbesartan in the treatment of diabetic nephropathy via the gut–kidney axis

**DOI:** 10.3389/fphar.2024.1424968

**Published:** 2024-10-28

**Authors:** Hongmei Yu, Haitao Tang, Rengui Saxu, Yuhui Song, Xu Cui, Jingjing Xu, Nan Li, Siyuan Cui, Haitao Ge, Wei Tang, Harvest F. Gu

**Affiliations:** ^1^ Laboratory of Molecular Medicine, School of Basic Medicine and Clinical Pharmacy, China Pharmaceutical University, Nanjing, Jiangsu Province, China; ^2^ Suzhong Pharmaceutical Group Co. Ltd., Suzhong Pharmaceutical Research Institute, Nanjing, Jiangsu Province, China; ^3^ Department of Endocrinology, Jiangsu Province Hospital of Traditional Chinese Medicine, The Affiliated Hospital of Nanjing University of Chinese Medicine, Nanjing, China; ^4^ Department of Endocrinology, Wuxi Second People’s Hospital, Jiangnan University Medical Center, Wuxi, Jiangsu Province, China; ^5^ Islet Cell Senescence and Function Research Laboratory, Department of Endocrinology, Nanjing Medical University Affiliated Geriatric Hospital/Jiangsu Province Geriatric Hospital, Nanjing, Jiangsu Province, China

**Keywords:** *Abelmoschus manihot* L., diabetic nephropathy, Huangkui capsule, irbesartan, type 2 diabetes

## Abstract

**Background:**

Clinical observations have recently shown that *Abelmoschus manihot* (L.) in the form of Huangkui capsule (HKC) and in combination with irbesartan (EB) is an effective therapy for diabetic nephropathy (DN) in patients with type 2 diabetes (T2D). The present study aims to explore the mechanisms underlying the therapeutic efficacies of HKC and its combination with EB in DN via the gut-kidney axis.

**Methods:**

HKC, EB, and their combination or vehicle were administered in db/db mice, which is an animal model for the study of T2D and DN. Comparative analyses of the gut microbiota, serum metabolites, and kidney transcriptomics before and after drug administration were performed.

**Results:**

After treatment with HKC, EB, and their combination for 4 weeks, the urinary albumin-to-creatinine ratios decreased significantly in the db/db mice with DN. In terms of the gut microbiota, the abundances of Faecalitalea, Blautia, and Streptococcus increased but those of Bacteroidetes, Firmicutes, Enterobacteriaceae, and Desulfovibrio decreased. Parallelly, serum metabolites, mainly including quercetin 3′-glucuronide and L-dopa, were elevated while cortisol and cytochalasin B were reduced. Furthermore, the *S100a8*, *S100a9*, *Trem1*, and *Mmp7* genes in the kidneys were downregulated. These altered elements were associated with proteinuria/albuminuria reduction. However, EB had no effects on the changes in blood pressure and specific differentially expressed genes in the kidneys.

**Conclusion:**

The present study provides experimental evidence that HKC regulates the gut microbiota, circulating metabolites, and renal gene activities, which are useful for better understanding of the action mechanisms of *A. manihot* in the treatment of DN through the gut-kidney axis.

## Introduction

*Abelmoschus manihot* (L.) is called Huangkui in Chinese (“Huang” means yellow and “Kui” means sunflower). Similar to the discovery of Artemisinin (Tu, 2016), the medical applications of *A. manihot* were first recorded in the Handbook of Prescriptions for Emergencies by Mr. Hong Ge during the Eastern Jin Dynasty (317–420 AD) in China. As a traditional Chinese medicine, the Huangkui capsule (HKC) is made from the ethanolic extract of the flowers of *A. manihot* and received approval from the Chinese Food and Drug Administration (Z19990040) in 1999 (Chen et al., 2016; Li et al., 2021). Currently, HKC is used in China for treating patients with kidney diseases, including diabetic nephropathy (DN). The main active chemical constituents of HKC are the flavones of *A. manihot* (L.); according to liquid chromatography quadrupole time-of-flight mass spectrometry (LC-Q-TOF/MS) analysis, we identified seven flavonoids as the components of HKC, namely rutin, hyperoside, isoquercitrin, gossypetin-8-O-β-D-glucuronide, myricetin, quercetin-3-O-β-D-glucuronide, and quercetin (Diao et al., 2024).

Clinical observations have demonstrated the efficacy and safety of HKC in the treatment of primary glomerular diseases and IgA nephropathy that mainly present as albuminuria and proteinuria (Zhang et al., 2014; Li et al., 2017; Li et al., 2020). Irbesartan (EB) is an angiotensin receptor blocker and that independently has renoprotective blood pressure lowering effects in patients with type 2 diabetes (T2D) and microalbuminuria (Parving et al., 2001; Lewis et al., 2001). When used in combination with EB, a multicenter randomized double-blind parallel controlled clinical trial recently reported HKC as an effective therapy for DN in T2D patients for reducing albuminuria and proteinuria (Zhao et al., 2022). However, the mechanisms underlying the effects of HKC and its combination with EB in the treatment of DN are unknown.

Gut microbiota and their metabolites may have pathogenic or beneficial effects on the onset and progression of DN. The theory of the gut-kidney axis was proposed by Meijers and Evenepoel (2011) based on their finding that changes in the intestinal microecology could affect the progression of chronic kidney disease by regulating metabolites (Meijers et al., 2019; Krautkramer et al., 2021). In recent years, increasing evidence has shown that gut microbiota and their metabolites play essential roles in the pathophysiological processes of DN through the gut-kidney axis (Zhao et al., 2023; Tao et al., 2019). Our research group has carried out an experimental study using non-obese diabetic (NOD) mice as a model for the study of type 1 diabetes and DN; the results suggest that HKC may modulate gut microbiota and subsequently ameliorate the metabolite levels in DN (Shi et al., 2023). Both HKC and EB are orally administered drugs that may exert their pharmaceutical effects in the treatment of DN by regulating the gut-kidney axis.

In the present work, we designed an experimental study using db/db mice as the animal model to study T2D and DN (Sharma et al., 2003; Wang et al., 2014). First, we investigated the changes in gut microbiota before and after administration of HKC, EB, and their combination. Then, we identified the altered metabolites in the serum. Finally, we analyzed the transcriptomics of the kidneys. This study provides novel information for understanding the efficacy of HKC and its combination with EB in the treatment of T2D-related DN with a focus on the gut-kidney axis.

## Methods

### Animals

Ten-week-old db/db (BKS.Cg-Dock7m +/+ Leprdb/J) and C57BL/KsJ mice were purchased from the animal experimental center of Nanjing University (Nanjing, China). All mice were males and housed in a specific pathogen-free barrier environment at the animal experimental center of Xuanwu campus, China Pharmaceutical University. The animal room was maintained at a temperature of 24°C ± 2°C and humidity of 50% ± 10% with 12-h light/dark cycles. After 1 week of adaptation, the bodyweights and blood glucose levels of all mice were measured weekly. The urine samples were collected using metabolic cages (DXL-XS, Fengshi, Suzhou, China) for 6 h. Microalbuminuria and creatinine were measured using ELISA quantitative kits (Elabscience Biotechnology, China). When the blood glucose level was ≥16.7 mmol/L and urinary albumin-to-creatinine ratio (UACR) was ≥200 ng/μg for two consecutive days, the db/db mice were diagnosed to have DN. The db/db mice with DN were randomly divided into five groups as DN without treatment (n = 13), HKC (receiving HKC treatment, n = 11), EB (receiving EB treatment, n = 6), HKCEB (receiving HKC with EB as treatment, n = 8), and C57BL/KsJ mice as the non-diabetic control group (WT, n = 16).

### Drugs and administration

HKC was produced by Suzhong Pharmaceutical Group Co., Ltd. (Taizhou, China). Each HKC contains 0.43 g of *A. manihot* (L.) extract. The quality of the HKC was examined through fingerprint analysis using high-performance liquid chromatography, as reported previously (Lai et al., 2009; Guo et al., 2015). EB was produced by Sanofi Shengdelabao Minsheng Pharmaceutical Co., Ltd. (Hangzhou, China). Based on the conversion of human and mouse body surface areas, HKC (0.84 g/kg/d) and EB (0.0195 g/kg/d) or vehicle were administered daily via oral gavage for 4 weeks in the db/db mice. The administration period was determined based on clinical trials (Zhao et al., 2022) and our previous experimental studies (Yu et al., 2023a; Yu et al., 2023b; Shi et al., 2023). The experiments were performed according to the guidelines of the Declaration of Helsinki and approved by the ethics committee of China Pharmaceutical University (Approval Code: 2019-08-0003 and Approval Date: 26-08-2019).

### 16S ribosomal DNA sequence analysis

As reported previously, our research group used the intestinal contents as samples to analyze the gut microbiota in mice (Shi et al., 2023). In the present study, mice colon were retrieved by surgical double ligation. Total g.DNA from the colon microbiota was extracted using the CTAB/SDS method and amplified by polymerase chain reaction (PCR) using 341F (5′-CCTAYGGGRBGCASCAG-3′) and 806R (5′-GGACTACNNGGGTATCTAAT-3′) as the primers belonging to the V3–V4 variable region of 16S rDNA. Each sample was repeated thrice, and the mixed PCR products were detected through 2% agarose gel electrophoresis. The samples with main band brightness between 400 and 450 bp were selected for further experiments. The amplicons were purified using the AxyPrep DNA gel extraction kit (Axygen Biosciences, Union City, CA, United States). After the DNA libraries were built using the NEB Ultra DNA Library Prep Kit (NEB), the 16S RNA sequences were aligned and analyzed using the NovaSeq 6000 (Illumina, San Diego, CA, United States) platform and Silva database.

### Serum metabolomics

Serum samples stored at −80°C were removed from the refrigerator and thawed on ice until there were no ice cubes in the samples. The samples were then mixed by vortexing for 10 s; approximately 50 μL of each sample was transferred to the corresponding numbered centrifuge tube, and 300 µL of a mixture of acetonitrile and methanol (ACN: methanol = 1:4, V/V) was added. The sample plates were vortexed for 3 min and centrifuged at 12,000 r/min for 10 min at 4°C. After centrifugation, approximately 200 μL of the supernatant was removed into another corresponding numbered centrifuge tube and placed in a refrigerator at −20°C for 30 min. Qualitative analysis of the serum metabolites was initially performed by untargeted metabolomics in the LC-Q-TOF/MS platform and further adapted to widely targeted metabolomics on the LC-ESI-MS/MS system. Acquisition conditions for the untargeted assays included ultraperformance liquid chromatography (UPLC) and quadrupole time-of-flight (TripleTOF 6600, AB SCIEX) data acquisition systems, and the targeted acquisition conditions included UPLC and tandem mass spectrometry (Xing et al., 2024a).

### Kidney transcriptomics

The total RNAs in the kidneys were extracted using a TRIzol regent kit (Ambion, Shanghai Yubo Biological Technology Co., Ltd., China). The concentration of the total RNAs was detected with the Qubit®RNA assay kit (Life Technologies, CA, United States). The integrity of the total RNAs was analyzed with the Nano6000 bioanalyzer 2100 system (Agilent Technologies, CA, United States). Library construction for the total RNAs >1 μg was performed with the NEBNext^®^ Ultra RNA library prep kit (Illumina, NEB, United States). Oligo (dT) beads were enriched using mRNA with polyA tail, and the mRNA were randomly interrupted in the NEB fragmentation buffer. The cDNA was synthesized using the M-MuLV reverse transcriptase system (Illumina, NEB, United States) with fragmented mRNA as the template and random oligonucleotides as the primers. The cDNAs of approximately 200 bp were screened using AMPure XP beads (Beckman Coulter, Beverly, United States). The library was quantitated using the Qubit2.0 Fluorometer, and the insert size was detected using the Agilent 5400 bioanalyzer (Agilent, United States). Once qualified, the library was sequenced on the Illumina platform (Illumina, Novo Geno Bioinformatics Co., Ltd., China).

### Statistical analysis

All analyses and graphics were obtained using GraphPad Prism software (version 8.0), SPSS software (version 23.0), and R software (version 3.6.0). The principal coordinates analysis (PCoA) was used for Adonis’s multivariate analysis of variance; linear discriminant analysis effect size (LEfSe) differences between pairs of groups were tested for significance using the Kruskal–Wallis sum-rank test. Biological significance was subsequently analyzed through pairwise tests among the groups using the Wilcoxon rank-sum test. Furthermore, the genus levels of the gut microbiota were used in the Metastats statistical analysis. Metabolic pathway analysis that integrated pathway enrichment and pathway topology analyses were visualized using MetaboAnalyst (Chong and Xia, 2020; Pang et al., 2022). The correlations among the gut microbiota, metabolites, and differentially expressed genes (DEGs) were analyzed using the Spearman correlation test. The significance comparisons between the groups were calculated using Student's t-test. All values are presented in terms of mean ± standard error of the mean (SEM) unless otherwise noted. A *p*-value < 0.05 was used as the threshold for statistical significance.

## Results

### UACR after treatment with HKC, EB, and their combination

**FIGURE 1:**
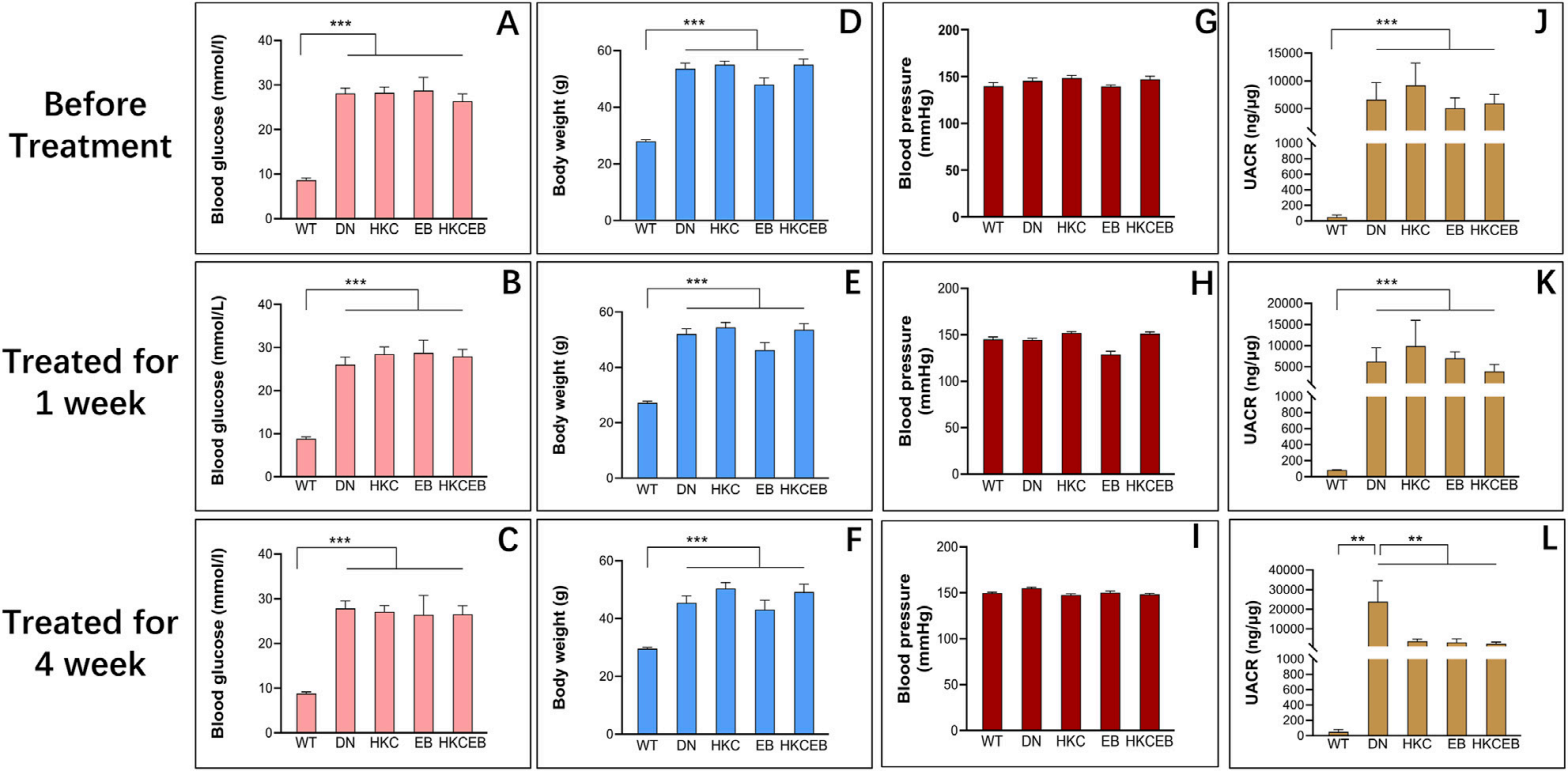
Blood glucose levels, bodyweights, UACRs, and blood pressure values of db/db mice before and after treatment with *Abelmoschus manihot*, irbesartan, and their combination. **(A–C)** Blood glucose, **(D–F)** bodyweight, **(G–I)** blood pressure, and **(J–L)** UACR of the DN group compared to the WT group and after treatment with HKC, EB, or HKCEB for 1 and 4 weeks. DN, diabetic nephropathy; WT, non-diabetic control; HKC, Huangkui capsule of *A. manihot*; EB, irbesartan; HKCEB, HKC combined with EB; UACR, urinary albumin-to-creatinine ratio; **P < 0.01 and ***P < 0.001.

The blood glucose, bodyweight, and UACR values of the db/db mice were higher than those of the mice in the WT group. After treatment with HKC, EB, and HKCEB in the db/db mice, the UACR decreased significantly (Figures 1J–L). As reported recently, several biomarkers such as col4a3, slc5a2, slc34a1, slc12a3, and slc4a1in the glomerulus as well as the proximal and distal convoluted tubules of the kidneys in db/db mice treated with HKC were observed to have improved (Yu et al., 2023a). However, changes in the blood glucose levels and bodyweights were not statistically significant (Figures 1A–F). The blood pressures of all mice were examined, and no significant changes were observed (Figures 1G–I).

### Changes in intestinal flora after treatment with HKC, EB, and their combination

**FIGURE 2:**
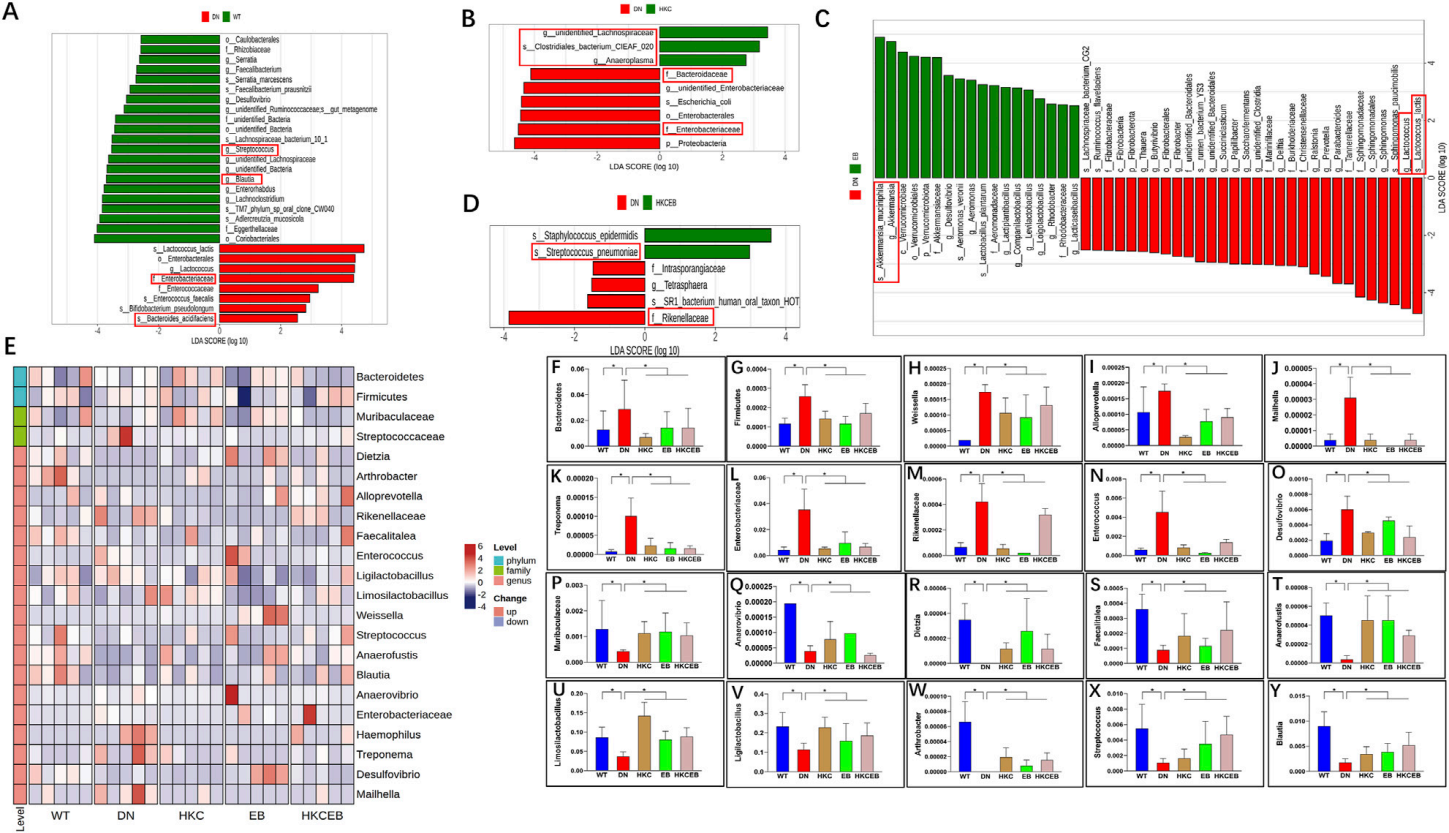
Changes in the intestinal flora after treatment with *A. manihot*, irbesartan, and their combination. LEfSe analyses demonstrate changes in the intestinal flora **(A)** in DN compared to the WT group and after the treatment with **(B)** HKC, **(C)** EB, and **(D)** HKCEB. **(E)** Clustering heatmap of the abundance of intestinal flora. The heatmap indicates that **(F–O)** 10 gut microbiota types were decreased while **(P–Y)** 10 other types of intestinal flora were increased after treatment with HKC, EB, or HKCEB. DN, diabetic nephropathy; WT, non-diabetic control; HKC, Huangkui capsule of *A. manihot*; EB, irbesartan; HKCEB, HKC combined with EB; *P< 0.05.

Comparative analyses of the intestinal flora in the WT, DN, HKC, EB, and HKCEB groups were conducted. A plot of the PCoA results is shown in Supplementary Figure S1A. Bacteroidetes and Firmicutes were the dominant phyla (Supplementary Figure S1B), while Ligilactobacillus and Limosilactobacillus were dominant at the genus level (Supplementary Figure S1F). LEfSe demonstrated that f._Enterobacteriaceae and s._Bacteroides_acidifaciens increased, while g._Streptococcus and g._Blautia decreased in DN compared to the WT group (Figure 2A). After HKC treatment, g._Anaeroplasma, g._unidentified_Lachnospiraceae, and s._Clostridiales_bacterium_CIEAF_020 increased (Figure 2B). In the EB group, g._Akkermansia was found to have increased (Figure 2C). s._Streptococcus_pneumoniae and s._Staphylococcus_epidermidis had increased in the HKCEB group (Figure 2D). A heatmap of the flora abundance of the gut microbiota in the WT, DN, HKC, EB, and HKCEB groups is shown in Figure 2E. The data demonstrate that Bacteroidetes, Firmicutes, Weissella, Alloprevotella, Mailhella, Treponema, Enterobacteriaceae, Rikenellaceae, Enterococcus, and Desulfovibrio increased in DN compared to WT but decreased after HKC, EB, and HKCEB treatments (Figures 2F–O). Conversely, Muribaculaceae, Anaerovibrio, Dietzia, Faecalitalea, Anaerofustis, *Limosilactobacillus*, *Ligilactobacillus*, *Arthrobacter*, *Streptococcus*, and Blautia were lower in DN but increased upon treatment with HKC, EB, and HKCEB (Figures 2P–Y).

### Alteration of serum metabolites after treatment with HKC, EB, and their combination

**FIGURE 3:**
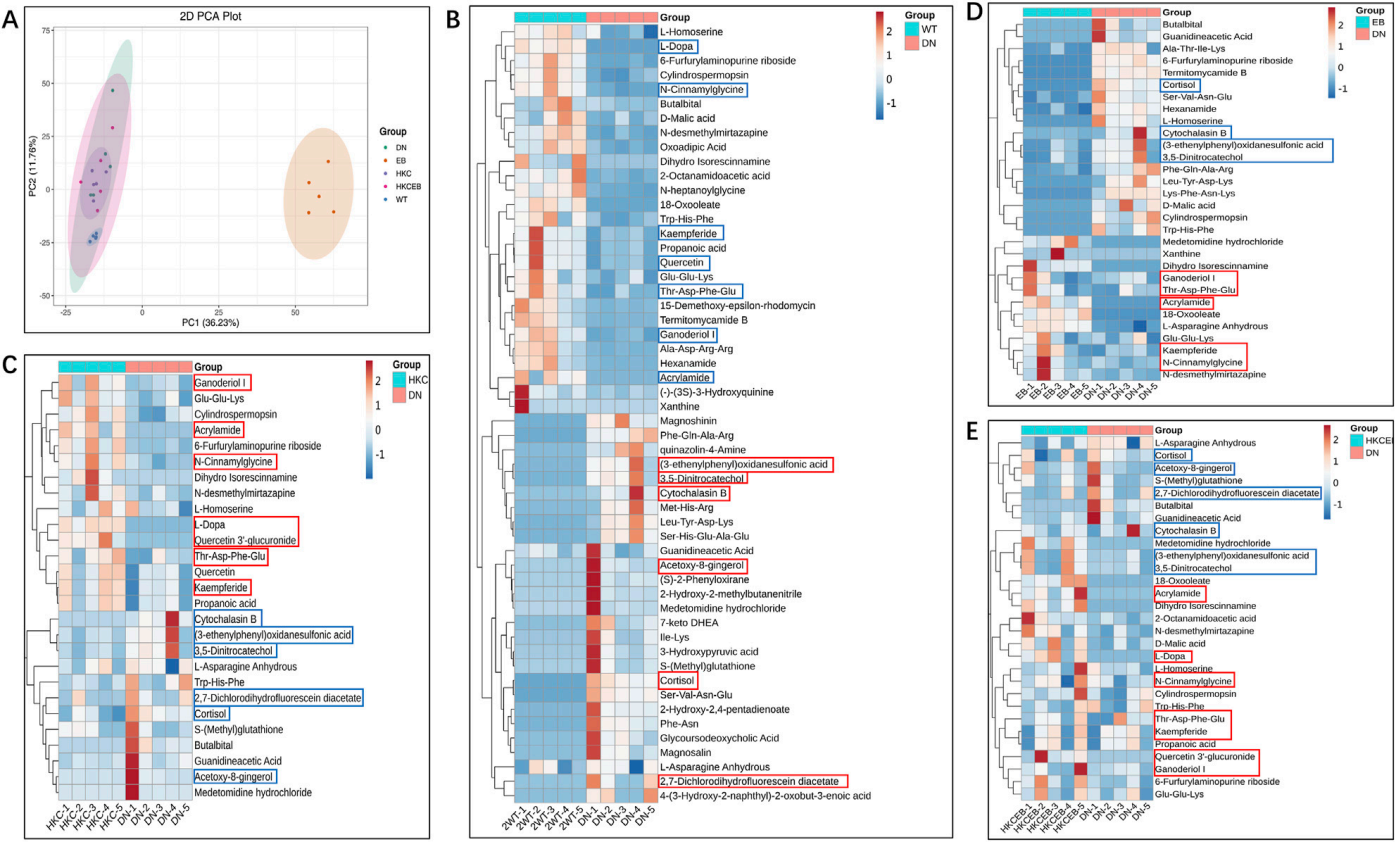
Alteration of serum metabolites after treatment with *A. manihot*, irbesartan, and their combination. **(A)** PCA plot of the altered serum metabolites in the WT, DN, HKC, EB, and HKCEB groups. **(B–E)** Cluster heatmaps of the top metabolites in DN compared to the WT group and after treatment with HKC, EB, or HKCEB. The red boxes indicate upregulation, while the blue boxes imply downregulation. PCA, principal component analysis; DN, diabetic nephropathy; WT, non-diabetic control; HKC, Huangkui capsule of *A. manihot*; EB, irbesartan; HKCEB, HKC combined with EB.

The numbers of metabolites detected in the sera of the WT, DN, HKC, EB, and HKCEB groups are summarized in Supplementary Figures S2A–C. Principal component analysis (PCA) shows the altered serum metabolites in the db/db mice after treatment with HKC, EB, and their combination (Figure 3A). The cluster heatmaps of the top-50 metabolites in DN and the remaining groups are shown in Figure 3B and Supplementary Figures S3A–C, respectively. Compared to the WT group, cortisol, cytochalasin B, acetoxy-8-gingerol, (3-ethenylphenyl) oxidanesulfonic acid, 3,5-dinitrocatechol, and 2,7-dichlorodihydrofluorescein diacetate were higher in the DN group (red boxes indicate upregulation), while kaempferide, quercetin, Thr-Asp-Phe-Glu, N-cinnamylglycine, ganoderiol I, L-dopa, and acrylamide were lower (blue boxes indicate downregulation). After HKC and HKCEB treatments, cortisol, cytochalasin B, 3,5-dinitrocatechol, (3-ethenylphenyl) oxidanesulfonic acid, 2,7-dichlorodihydrofluorescein, and acetoxy-8-gingerol were found to be downregulated, while kaempferide, ganoderiol I, Thr-Asp-Phe-Glu, N-cinnamylglycine, and acrylamide were upregulated (Figures 3C–E). In the EB group, only cortisol, cytochalasin B, 3,5-dinitrocatechol, and (3-ethenylphenyl) oxidanesulfonic acid were found to be downregulated, while kaempferide, ganoderiol I, Thr-Asp-Phe-Glu, and N-cinnamylglycine were upregulated (Figure 3E).

### KEGG pathway enrichment analyses of the metabolites

**FIGURE 4:**
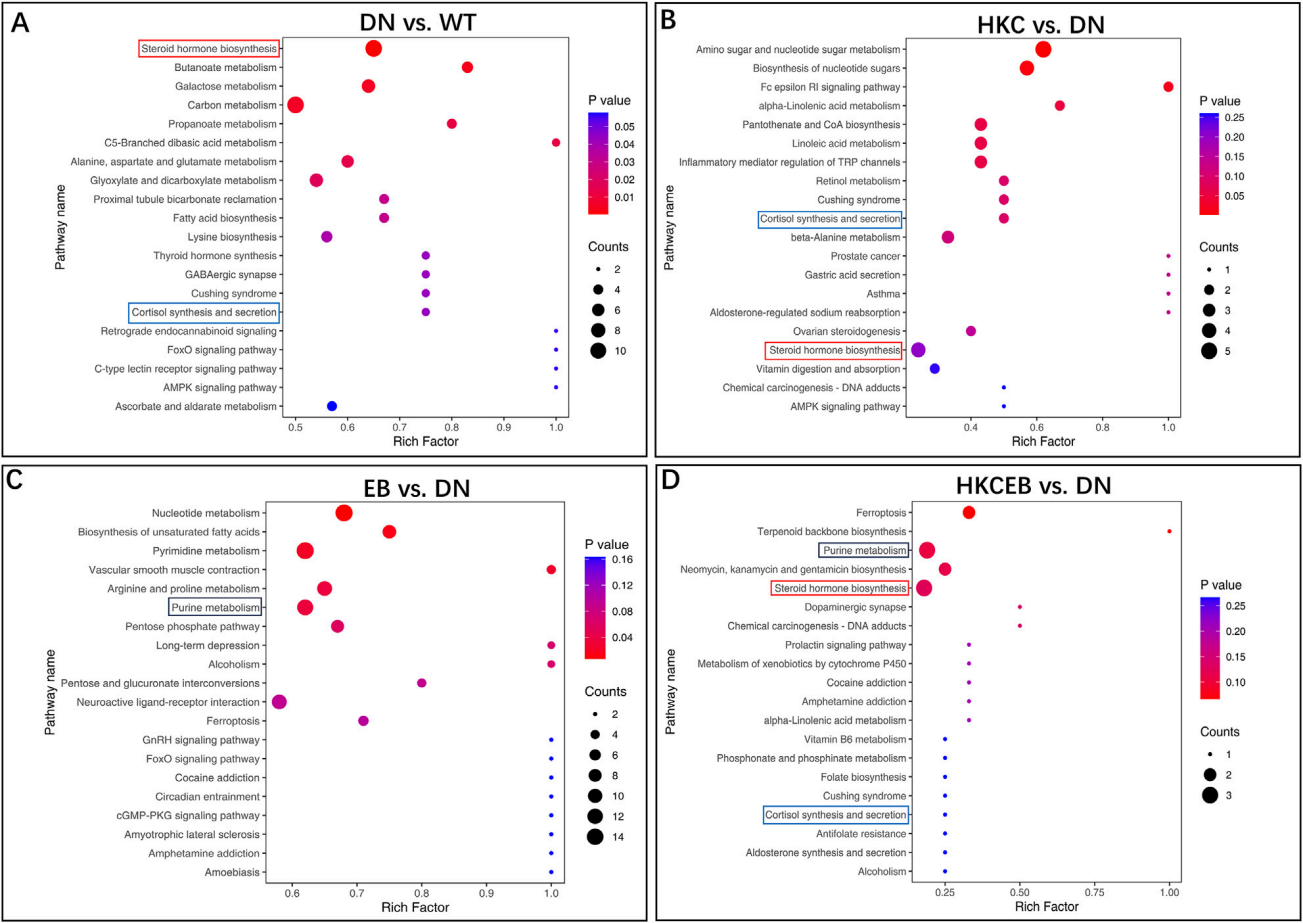
KEGG pathway enrichment analyses of the various metabolites. The predicted KEGG pathways of the different serum metabolites **(A)** in DN compared to WT and **(B–D)** after treatment with HKC, EB, or HKCEB are presented. DN, diabetic nephropathy; WT, non-diabetic control; HKC, Huangkui capsule of *A. manihot*; EB, irbesartan; HKCEB, HKC combined with EB.

The Kyoto encyclopedia of genes and genomes (KEGG) is a database for systematic analysis of the gene functions and genome information; KEGG also provides integrated metabolic pathways for carbohydrates, nucleosides, and amino acids. The annotation results of the significantly altered metabolites in the DN, HKC, EB, and HKCEB groups are shown in Supplementary Figure S4A–D and subsequently classified into four major KEGG pathways, including organismal systems, metabolism, human disease, and environmental information processing and cellular processing (Supplementary Figure S5A–D). In the DN group, cortisol was found in the steroid hormone biosynthesis, cortisol synthesis, and secretion pathways; 3-hydroxypyruvic acid was enriched in the carbon metabolism, glyoxylate, and dicarboxylate metabolism pathways (Figure 4A). In the HKC group, cortisol was downregulated and enriched in the steroid hormone biosynthesis as well as cortisol synthesis and secretion pathways (Figure 4B). In the EB group, hypoxanthine, xanthine, erucic acid, L-arginine, and urea were found to be enriched in the nucleotide and purine metabolism pathways. Moreover, erucic acid was downregulated in the nucleotide metabolism pathway, while L-arginine was upregulated and enriched in the arginine and proline metabolism pathways. Urea was downregulated in the pyrimidine metabolism pathway (Figure 4C). In the HKCEB group, 11β-hydroxyprogesterone, 17α-hydroxyprogesterone, and xanthosine were found in the steroid hormone biosynthesis and purine metabolism pathways (Figure 4D).

### Correlation between gut microbiota changes and serum metabolite alterations

**FIGURE 5:**
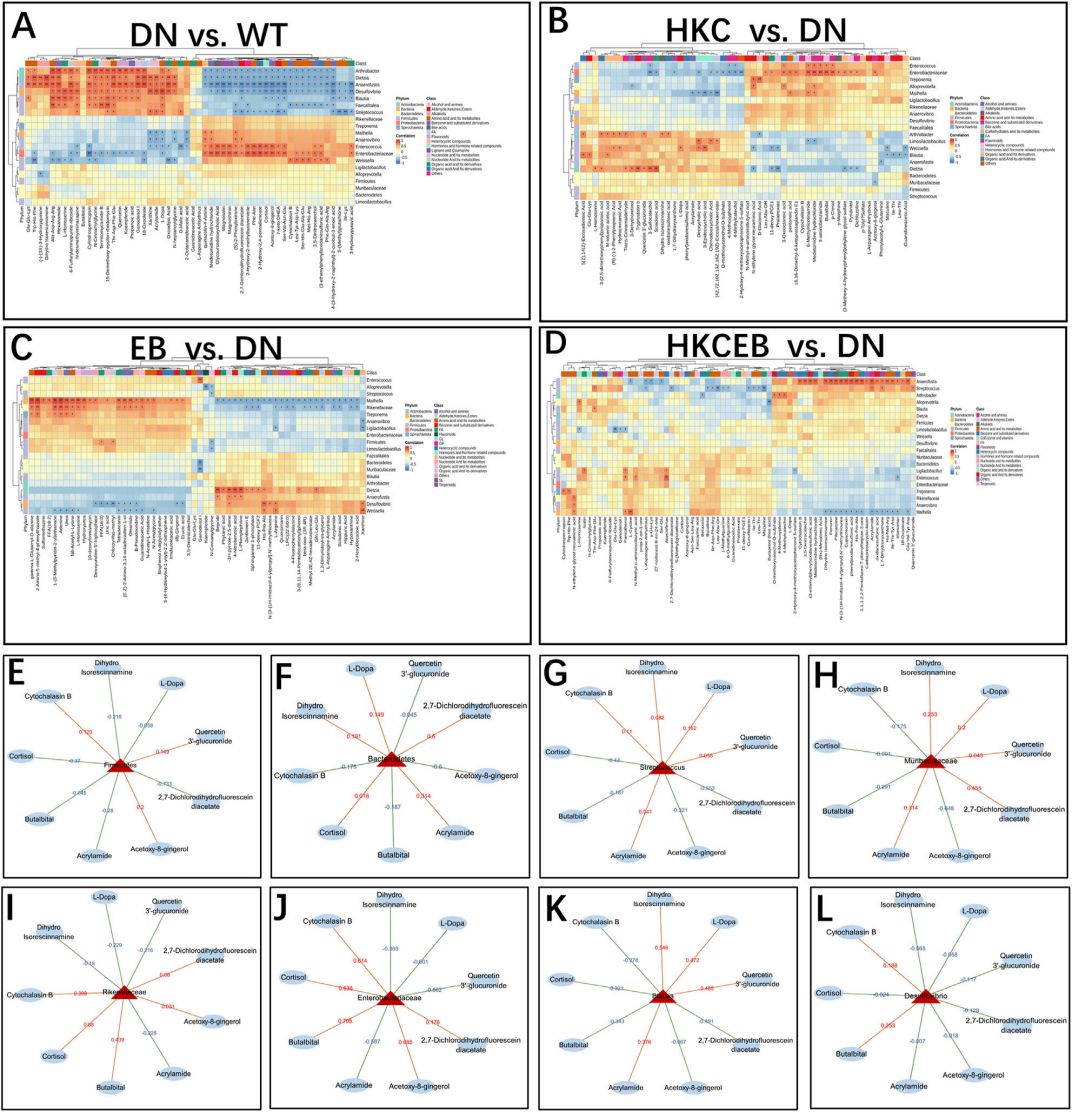
Correlation between changes to the gut microbiota and various serum metabolites **(A)** in DN compared to the WT group and **(B–D)** after treatment with HKC, EB, or HKCEB. **(E–L)** The major gut microbiota are shown, and each is correlated with several serum metabolites. The red lines indicate positive correlation while the blue lines indicate negative correlation. DN, diabetic nephropathy; WT, non-diabetic control; HKC, Huangkui capsule of *A. manihot*; EB, irbesartan; HKCEB, HKC combined with EB.

The correlations between metabolites and gut microbiota at the phylum, family, and genus levels in DN compared to WT and after treatment with HKC, EB, and HKCEB are shown in Figures 5A–D. At the phylum level, Firmicutes was positively (indicated by red line) correlated with cytochalasin B and quercetin 3′-glucuronide but negatively (blue line) correlated with L-dopa and cortisol, while Bacteroidetes showed converse trends to Firmicutes (Figures 5E, F). At the family level, Muribaculaceae and Streptococcus were positively correlated with L-dopa, quercetin 3′-glucuronide, and acrylamide but negatively correlated with cortisol and acetoxy-8-gingerol (Figures 5G, H). At the genus level, Rikenellaceae and Enterobacteriaceae were positively correlated with cytochalasin B, cortisol, and acetoxy-8-gingerol but negatively correlated with L-dopa, quercetin 3′-glucuronide, and acrylamide (Figures 5I, J). Blautia was positively correlated with L-dopa, quercetin 3′-glucuronide, and acrylamide but negatively correlated with cytochalasin B, cortisol, and acetoxy-8-gingerol (Figure 5K). Desulfovibrio was positively correlated with cytochalasin B and negatively correlated with L-dopa, quercetin 3′-glucuronide, acrylamide, and acetoxy-8-gingerol (Figure 5L).

### DEGs in kidneys after treatment with HKC, EB, and their combination

**FIGURE 6:**
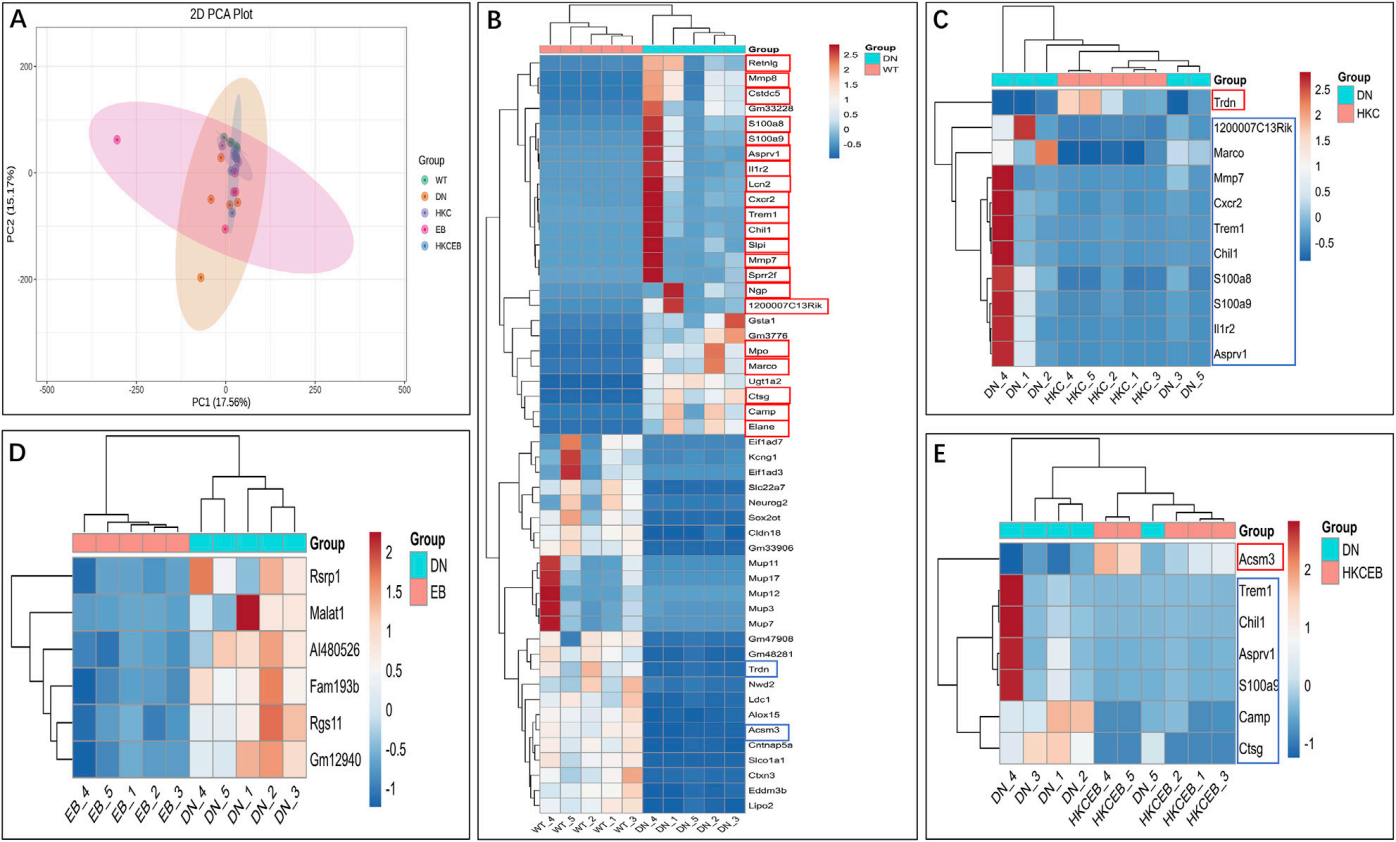
Cluster heatmap analyses of the differentially expressed genes in the kidneys. **(A)** PCoA plot and **(B–E)** cluster heatmaps show the differentially expressed genes in the kidneys of DN group compared to the WT group and after treatment with HKC, EB, or HKCEB. The red boxes indicate upregulation, while the blue boxes indicate downregulation. PCoA, principal coordinates analysis; DN, diabetic nephropathy; WT, non-diabetic control; HKC, Huangkui capsule of *A. manihot*; EB, irbesartan; HKCEB, HKC combined with EB.

PCoA implicated that the DEGs in the kidneys of the DN mice may be regulated after HKC, EB, and HKCEB treatments (Figure 6A). The DEGs in the kidneys of the DN group compared to the WT, HKC, EB, and HKCEB groups are summarized by Venn diagrams (Supplementary Figures S6A–C). The top-50 genes in the kidneys of the DN, HKC, EB, and HKCEB groups are represented as cluster heatmaps (Figures 6B–E; Supplementary Figures S7A–C). In brief, the *Cstdc5*, *S100a8*, *S100a9*, *Asprv1*, *Il1r2*, *Lcn2*, *Cxcr2*, *Trem1*, *Chil1*, *Slpi*, *Mmp7*, *Sprr2f*, *Ngp*, *1200007C13Rik*, *Mpo*, *Marco*, *Camp*, *Ctsg*, and *Elane* genes were upregulated, while the *Trdn* and *Acsm3* genes were downregulated in the DN group (Figure 6B). In the HKC group, *Trdn* was upregulated, while *Trem1*, *S100a8*, *Mmp7*, *S100a9*, *Asprv1*, *Chil1*, *Il1r2*, *1200007C13Rik*, *Cxcr2*, and *Marco* were downregulated (Figure 6C). In the EB group, all *Fam193b*, *Malat1*, *Rgs11*, *Gm12940*, *Rsrp1*, and *AI480526* genes were downregulated (Figure 6D). In the HKCEB group, *Acsm3* gene expression increased while *Trem1*, *Camp*, *S100a9*, *Asprv1*, *Chil1*, and *Ctsg* gene expressions decreased (Figure 6E).

### KEGG pathway enrichment analyses of the DEGs

**FIGURE 7:**
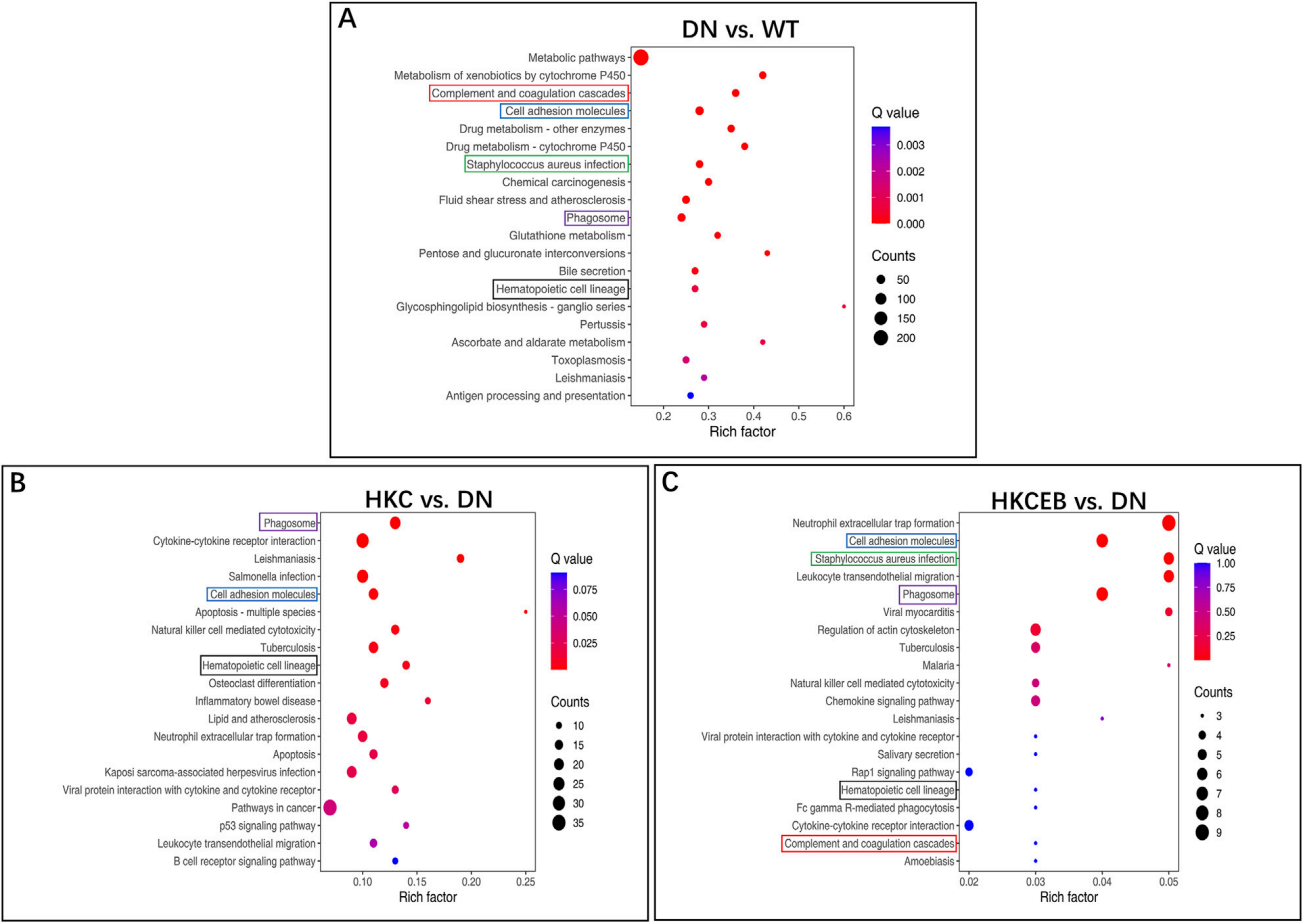
KEGG pathway enrichment analysis of the differentially expressed genes in the kidneys **(A)** for DN compared to the WT group and **(B, C)** after treatment with HKC or HKCEB. DN, diabetic nephropathy; WT, non-diabetic control; HKC, Huangkui capsule of *A. manihot*; HKCEB, HKC combined with EB.

Volcanic map, gene ontology (GO), and KEGG enrichment analyses of the DEGs in the kidneys of the DN, HKC, EB, and HKCEB groups are shown in Supplementary Figures S8–S10. The genes *Gsta1* and *Ugt1a2* that were upregulated in DN were enriched in the glutathione, pentose, and glucuronate interconversion pathways (Figure 7A). After HKC treatment, *Tlr4* was downregulated and enriched in the inflammatory bowel disease pathway (Figure 7B). After HKCEB treatment, *Selp* and *Itgb2* were enriched in the neutrophil extracellular trap formation pathway (Figure 7C). No specific pathways of the DEGs in the kidneys of the EB group were found.

### Correlation between serum metabolites and renal genes

**FIGURE 8:**
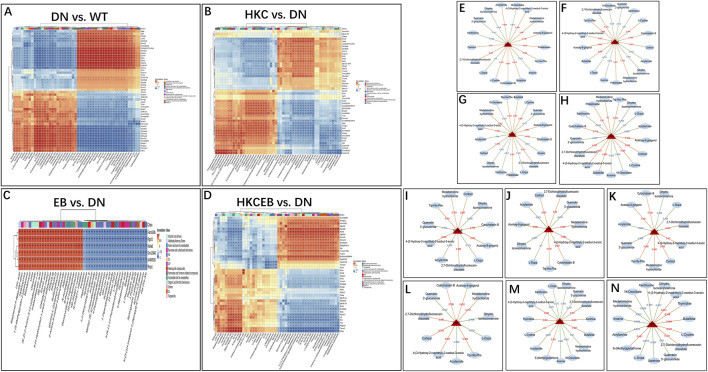
Correlation between serum metabolites and differentially expressed genes in the kidneys. **(A–D)** Heatmaps predicted by KEGG enrichment analysis show the correlations between the serum metabolites and renal genes in DN compared to WT and after treatment with HKC, EB, or HKCEB. **(E–N)** The genes expressed in the kidneys are functionally related to metabolites positively (red) or negatively (blue). DN, diabetic nephropathy; WT, non-diabetic control; HKC, Huangkui capsule of *A. manihot*; EB, irbesartan; HKCEB, HKC combined with EB.

KEGG enrichment bubble maps predicting the correlations between the serum metabolites and genes in the kidneys are shown in Supplementary Figures S11, S12. The 50 DEGs (only 6 in the EB group) and 50 differential metabolites were included in the Spearman correlate analyses, whose heatmaps are shown in Figures 8A–D. The *Trem1*, *S100a9*, *Asprv1*, and *Chil1* genes were upregulated in DN but downregulated in the HKC and HKCEB groups. These genes were positively correlated with metabolites such as cortisol, cytochalasin B, 2,7-dichlorodihydrofluorescein diacetate, medetomidine hydrochloride, and L-cystin but negatively correlated with dihydro isorescinnamine, L-dopa, 18-oxooleate, quercetin 3′-glucuronide, xanthosine, and 18-oxooleate (Figures 8E–H). In addition, the *S100a8*, *Mmp7*, *Il1r2*, and *Cxcr2* genes that were increased in DN but decreased after HKC treatment were positively correlated with cortisol, cytochalasin B, 2,7-dichlorodihydrofluorescein diacetate, medetomidine hydrochloride, and 4-(3-hydroxy-2-naphthyl)-2-oxobut-3-enoic acid but negatively correlated with dihydro isorescinnamine, L-dopa, quercetin 3′-glucuronide, and acrylamide (Figures 8I–L). In the HKCEB group, the *Camp* and *Ctsg* genes were positively correlated with 2,7-dichlorodihydrofluorescein diacetate, 4-(3-hydroxy-2-naphthyl)-2-oxobut-3-enoic acid, L-cystin, butalbital, S-(methyl) glutathione, and thymidine but negatively correlated with dihydro isorescinnamine, L-dopa, quercetin 3′-glucuronide, xanthosine, 18-oxooleate, medetomidine hydrochloride, and acrylamide (Figures 8M, N).

## Discussion

**FIGURE 9:**
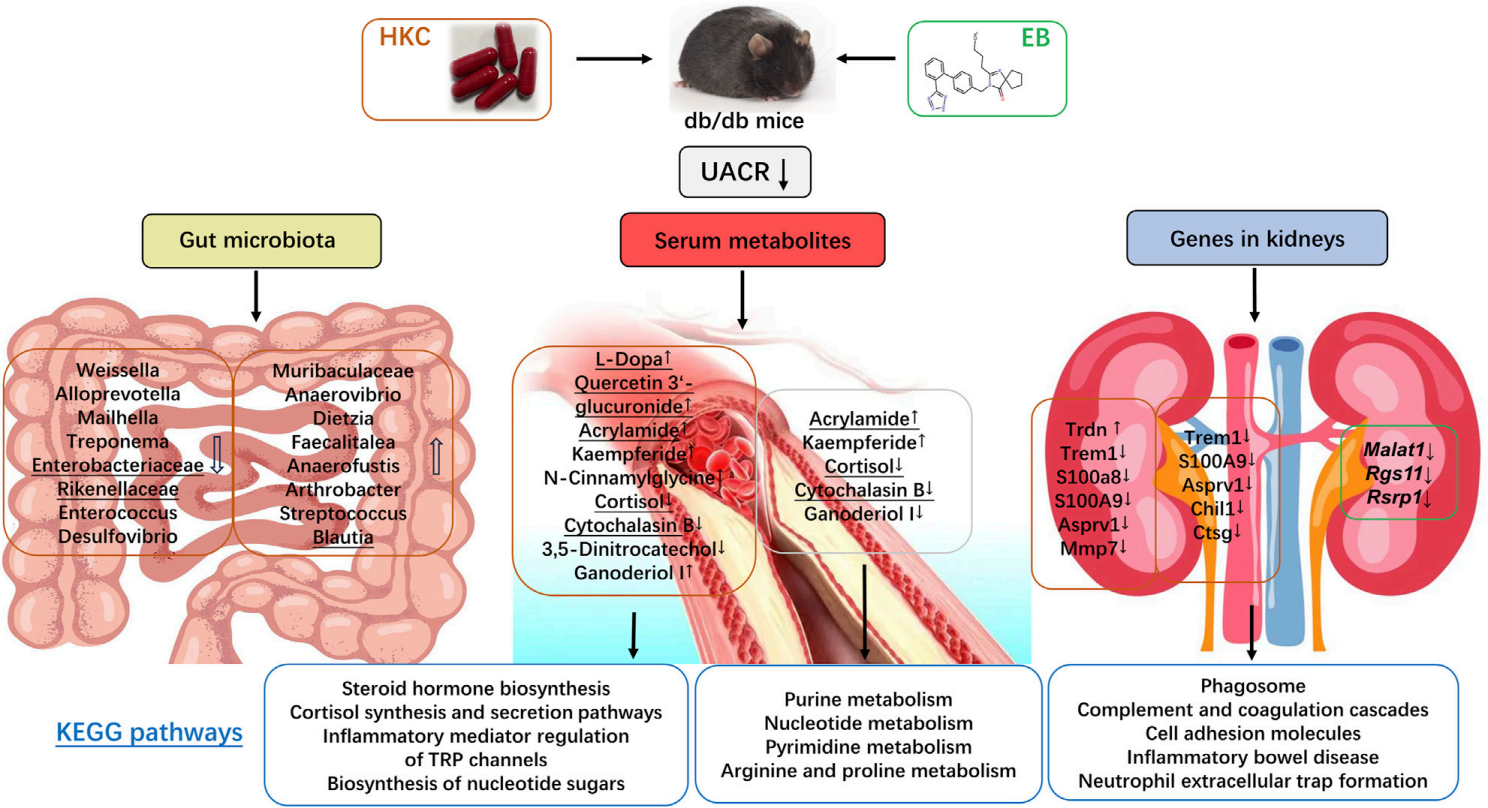
Abelmoschus manihot (L.) and its combination with irbesartan regulated the gut–kidney axis.

In the present study, we investigated the changes in the intestinal flora, serum metabolites, and mRNA expressions in the kidneys of db/db mice with DN. The aim here was to explore the molecular mechanisms of HKC and its combined treatment with EB for T2D-related DN primarily in the gut-kidney axis. After 4 weeks of administration of HKC, EB, and their combination, the UACRs of the db/db mice with DN were found to be significantly reduced, as reported in recent clinical observations (Zhao et al., 2022) and animal experiments (Yu et al., 2023a; Yu et al., 2023b). Furthermore, the molecular mechanisms of HKC and its combined treatment with EB for T2D-related DN were predicted from data analyses and summarized in Figure 9.

Based on analyses of the gut microbiota, we found that microorganisms like Alloprevotella, Bacteroides, Desulfovibrio, Enterobacteriaceae, Enterococcus, Firmicutes, Treponema, Rikenellaceae, and Weissella were increased in DN compared to the WT group, while Muribaculaceae, Anaerovibrio Ligilactobacillus, Limosilactobacillus, Faecalitalea were decreased. After treatment with HKC and HKCEB, however, these microbes underwent opposite changes, with the trends in DN transitioning from increasing to decreasing or from decreasing to increasing compared to those in WT. In recent years, increasing evidence has shown that microbiota are associated with diabetes and diabetic complications, including DN (Zhao et al., 2023; Tao et al., 2019). Gut microbiota such as Firmicutes and Bacteroidetes are found to be the dominant species in rats with T2D and DN, while Firmicutes and Bacteroides can be reduced by treatment with the San-Huang-Yi-Shen capsule to improve DN (Su et al., 2022). Weissella in the intestinal flora can be reduced using white common bean extract and subsequently ameliorate T2D and its complications (Feng et al., 2022). Muribaculaceae can be increased using the sodium-glucose cotransporter 2 inhibitors canagliflozin and dapagliflozin to prevent DN progression as well as the onset of end-stage renal disease independent of lowering glucose levels (Wu et al., 2023). Limosilactobacillus could decrease blood glucose levels in db/db mice and alleviate diabetes-mediated liver and kidney damage (Hsieh et al., 2020), so it has been considered as a probiotic in diabetic patients (Lacerda et al., 2022). Furthermore, Enterobacteriaceae, Blautia, and Rikenellaceae are found to be related to proteinuria and albuminuria in DN (Su et al., 2022; He et al., 2021). The Enterobacteriaceae, Enterococcus, and Desulfovibrio were significantly increased in the intestinal flora of patients with diabetic kidney disease, and Enterobacteriaceae was positively correlated with urinary proteins in fecal samples of adult patients with chronic kidney disease and idiopathic nephrotic syndrome (He et al., 2021).

In the analyses of the serum metabolites, several metabolites such as cortisol and cytochalasin B were found to be elevated in the DN group. After treatment with HKC and HKCEB, these two metabolites were regulated in opposite directions. Several clinical studies have shown that cortisol is higher in the plasma of patients with T2D-related DN (Devi et al., 2019). Serum cortisol levels in T2D patients and prediabetic subjects are elevated and associated with high levels of microalbuminuria (Zhang et al., 2020). Cytochalasin B is as a cytopermeable mycotoxin that can inhibit the loss of nephrin in the podocytes to reduce proteinuria (Doublier et al., 2003; Doublier et al., 2005). Furthermore, quercetin and L-dopa were found to be downregulated in the serum of db/db mice with DN. After treatment with HKC, quercetin 3′-glucuronide (the main metabolite of quercetin) was upregulated; however, this was not true for HKCEB. A previous study indicated that quercetin 3′-glucuronide can improve podocyte injury in DN rats by inhibiting oxidative stress and the TGF-β1/Smad pathway (Guo et al., 2013). Interestingly, L-dopa or 3,4-dihydroxyphenylalanine is a chiral amino acid generated via biosynthesis of L-tyrosine (Giuri et al., 2021); experimental studies have suggested that this small peptide can be used to induce hyperhomocysteinemia and therapeutically prevent the progression of Parkinson’s disease (Barthelmebs et al., 1991) while suppressing streptozotocin-induced diabetic glomerular hyperfiltration, subsequently preventing the progress of DN (Barthelmebs et al., 1990).

Furthermore, transcriptomics analyses demonstrated that genes such as *Trem1*, *S100a8*, *S100a9*, and *Mmp7* are higher in the kidneys of patients with DN compared to those in the WT group. The triggering receptor expressed on monocytes 1 (*Trem1*) gene amplifies neutrophil- and monocyte-mediated inflammatory responses triggered by bacterial and fungal infections by stimulating the release of proinflammatory chemokines and cytokines. As an amplifier of inflammation, *Trem1* has been shown to have a role in cIgA1-induced kidney injury (Zhao et al., 2018) and in maintaining tubular homeostasis through regulation of mitochondrial metabolic flexibility (Tammaro et al., 2019). The *S100a8* and *S100a9* genes encode myeloid-related protein 8 (MRP8) and migration inhibitory factor related protein 14 (MRP14), respectively; these two proteins are associated with immune and inflammation responses (Sreejit et al., 2020). Du et al. (2023) recently reported that the activities of S100A8 and S100A9 are increased in the tubular epithelial cells under DN. Kuwabara et al. previously reported that the *S100a8* gene expression in the glomeruli of the kidneys is associated with the progression of proteinuria and albuminuria in patients with obesity and T2D; they suggested that *S100a8* may induce inflammatory changes in macrophages via TLR4 signaling (Kuwabara et al., 2014). The matrix metalloproteinase 7 (*Mmp7*) gene expression is strongly correlated with fibrosis and with eGFR (Yu et al., 2023a; Yu et al., 2023b). Hirohama et al. (2023) recently conducted a proteomics analysis of kidney samples from patients with DN and identified *Mmp7* as a diagnostic marker of kidney fibrosis. Recent studies have shown that *A. manihot* polysaccharide (AMP) fortifies the intestinal mucus barrier by increasing mucus production, which plays a crucial role in AMP-mediated amelioration of colitis. The effects of AMP on mucus production are dependent on IL-10. These findings suggest that plant polysaccharides fortify the intestinal mucus barrier by maintaining homeostasis of the gut microbiome (Wang et al., 2024). MMP-7 is implicated in regulating kidney homeostasis and diseases and barely expressed in normal adult kidney but upregulated in acute kidney injury (AKI) and chronic kidney disease (CKD) (Liu et al., 2020). Decoction of white aconite (DWA) suppressed mRNA expression of fibrosis markers include Collagen I, CTGF, TGF-β, inhibited protein levels of MMP-9, α-SMA, and Galectin-3, while elevating TIMP1 expression (Xing et al., 2024b). Furthermore, Urine MMP7 as a kidney injury biomarker (Avello et al., 2023). In the present study, we found that these three genes were downregulated by HKC; furthermore, the *Trdn* gene expression in the DN group was lower than that in the WT group, which is a unique gene that is downregulated after treatment with HKC but not EB.

Based on the findings regarding the gut microbiota, serum metabolites, and gene activities in the kidneys, we conducted further correlation analysis and found that Enterobacteriaceae, Rikenellaceae, and Blautia were positively correlated with cortisol and cytochalasin B but negatively correlated with quercetin 3′-glucuronide and L-dopa. Furthermore, *Trem1*, *S100a8*, *S100a9*, and *Mmp7* were positively correlated with cortisol and cytochalasin B but negatively correlated with quercetin 3′-glucuronide, L-dopa, and dihydro isorescinnamine. All these related changes in the gut microbiota, circulating metabolites, and renal genes have been directly or indirectly proven to be associated with the reduction of urinary protein/albumin [24–45]. Therefore, the findings of the present study suggest that HKC combined with EB has multiple effects on the gut-kidney axis and that they could be involved in reducing inflammation, improving the functions of renal reabsorption and regulation, and delaying the progress of DN.

EB is clinically used in the treatment of essential hypertension and DN (Diao et al., 2024; Doublier et al., 2005). However, no significant effects were observed for EB on regulating blood pressure in db/db mice in this study. Although several serum metabolites were found to be associated with EB, no specific pathways were found for the DEGs in the kidneys. Su et al. (2022) recently reported the effects of the San-Huang-Yi-Shen capsule on rats with DN using EB as the control. Similarly, EB was not found to cause any significant improvements in the blood glucose level and renal function (Li et al., 2017). Given these findings, there are a few limitations to the present study. First, the main active chemical constituents of HKC were found to be rutin, hyperoside, isoquercitrin, gossypetin-8-O-β-D-glucuronide, myricetin, quercetin-3-O-β-D-glucuronide, and quercetin; among these, the flavonoid that has the primary effect on DN is unknown. Second, the plasma lipids and their metabolism are not included in the analyses. Third, female db/db mice were not included in the study because of changes in their estrogen and progesterone levels. Therefore, further investigation of the chemical constituents of *A. manihot* (L.) as well as the effects of HKC on plasma lipid metabolism and females are needed.

## Conclusion

The present study provides experimental evidence that *A. manihot* (L.) in the form of HKC has multiple effects on regulation of the gut-kidney axis. The data concerning changes to the gut microbiota, serum metabolites, and DEGs in the kidneys may be useful for improved understanding the mechanisms of HKC in the treatment of DN for reducing albuminuria and proteinuria.

## Data Availability

The datasets presented in this study can be found in online repositories. The names of the repository/repositories and accession number(s) can be found below: NCBI (accession: PRJNA1069301).

## Abbreviations

DN, diabetic nephropathy; DCT, distal convoluted tubule; DTC, distinct tubule cell; EB, irbesartan; ESRD, end-stage renal disease; GFR, glomerular filtration rate; HKC, Huangkui capsule; PCT, proximal convoluted tubule; PTC, proximal tubule cell; SLC, solute carrier; T2D, type 2 diabetes.
