# Supplementary material for: Effects of Abelmoschus manihot (L.) and its combination with irbesartan in the treatment of diabetic nephropathy via the gut–kidney axis
Source: Front Pharmacol. 2024 Oct 28;15:1424968. doi: 10.3389/fphar.2024.1424968 (PMC11550981; doi:10.3389/fphar.2024.1424968)
Supplement: Supplementary file 1 [file DataSheet1.PDF]

Supplemental Files

Yu Hongmei et al. Effects of Abelmoschus manihot (L.) and its Combination with Irbesartan in the Treatment of Diabetic Nephropathy via the Gut-Kidney Axis

Fig. S1 Comparative analyses of the intestinal flora

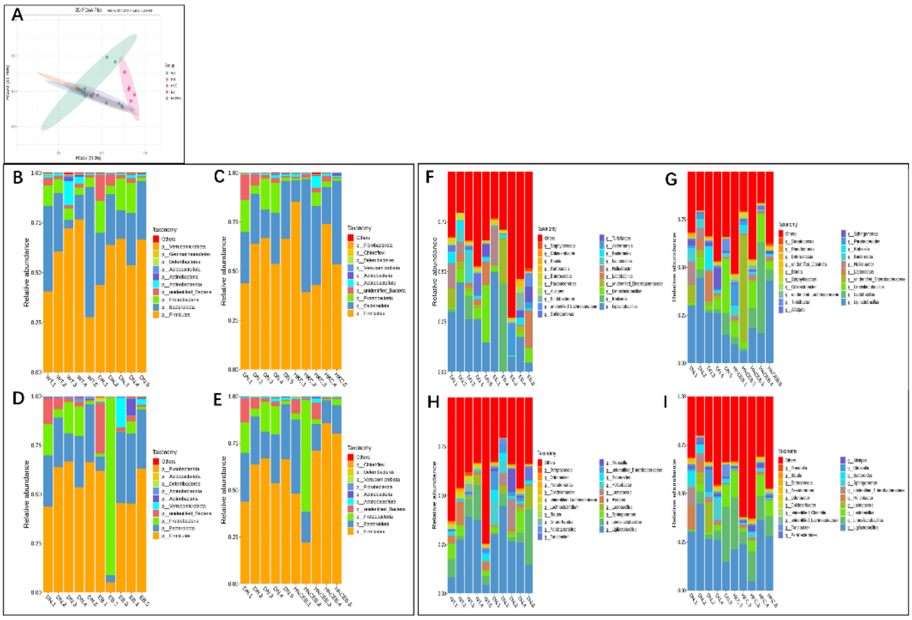

A plot of PCoA analysis of the intestinal flora in the groups of WT, DN, HKC, EB and HKCEB were represented (A). Bacteroidetes and Firmicutes were the dominant phyla at phylum levels (B-E), while Ligilactobacillus and Limosilactobacillus were the dominant phyla at genus levels (F-I). DN: diabetic nephropathy; WT: non-diabetic control; HKC: Huangkui capsule of *A. manihot*; EB: irbesartan; HKCEB: HKC combined with EB.

Fig. S2 The numbers of metabolites detected in serum

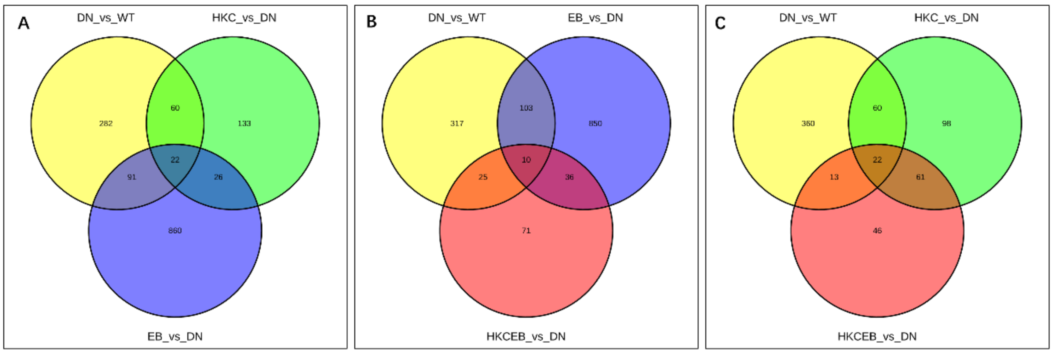

A total of 655 metabolites were detected in serum of db/db mice with DN, while 241, 999 and 142 metabolites were found in db/db mice of HKC, EB and HKCEB groups, respectively (A-C). DN: diabetic nephropathy; WT: non-diabetic control; HKC: Huangkui capsule of *A. manihot*; EB: irbesartan; HKCEB: HKC combined with EB.

Fig. S3 The cluster heatmap of TOP50 metabolites detected in serum

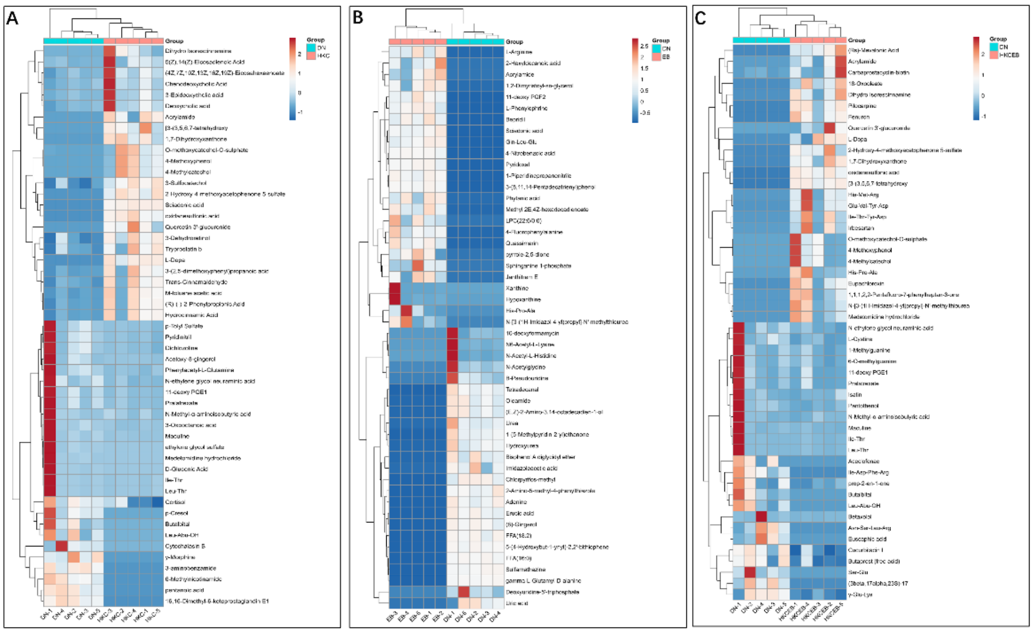

HKC: Huangkui capsule of *A. manihot*; EB: irbesartan; HKCEB: HKC combined with EB.

Fig. S4 Volcanic map of the changed metabolites in serum

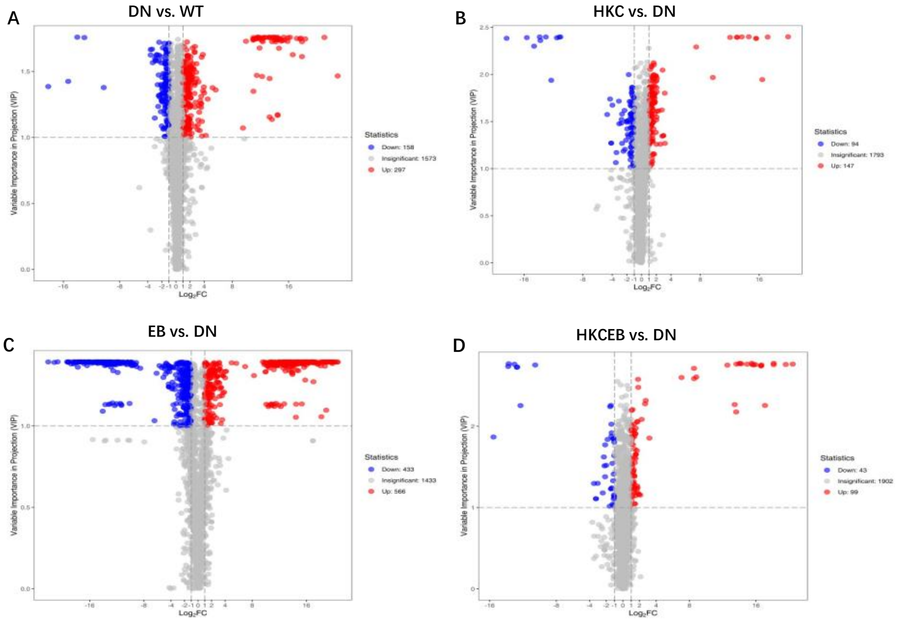

DN: diabetic nephropathy; WT: non-diabetic control; HKC: Huangkui capsule of *A. manihot*; EB: irbesartan; HKCEB: HKC combined with EB.

Fig. S5 Major pathways based upon the KEGG enrichment analyses of metabolites

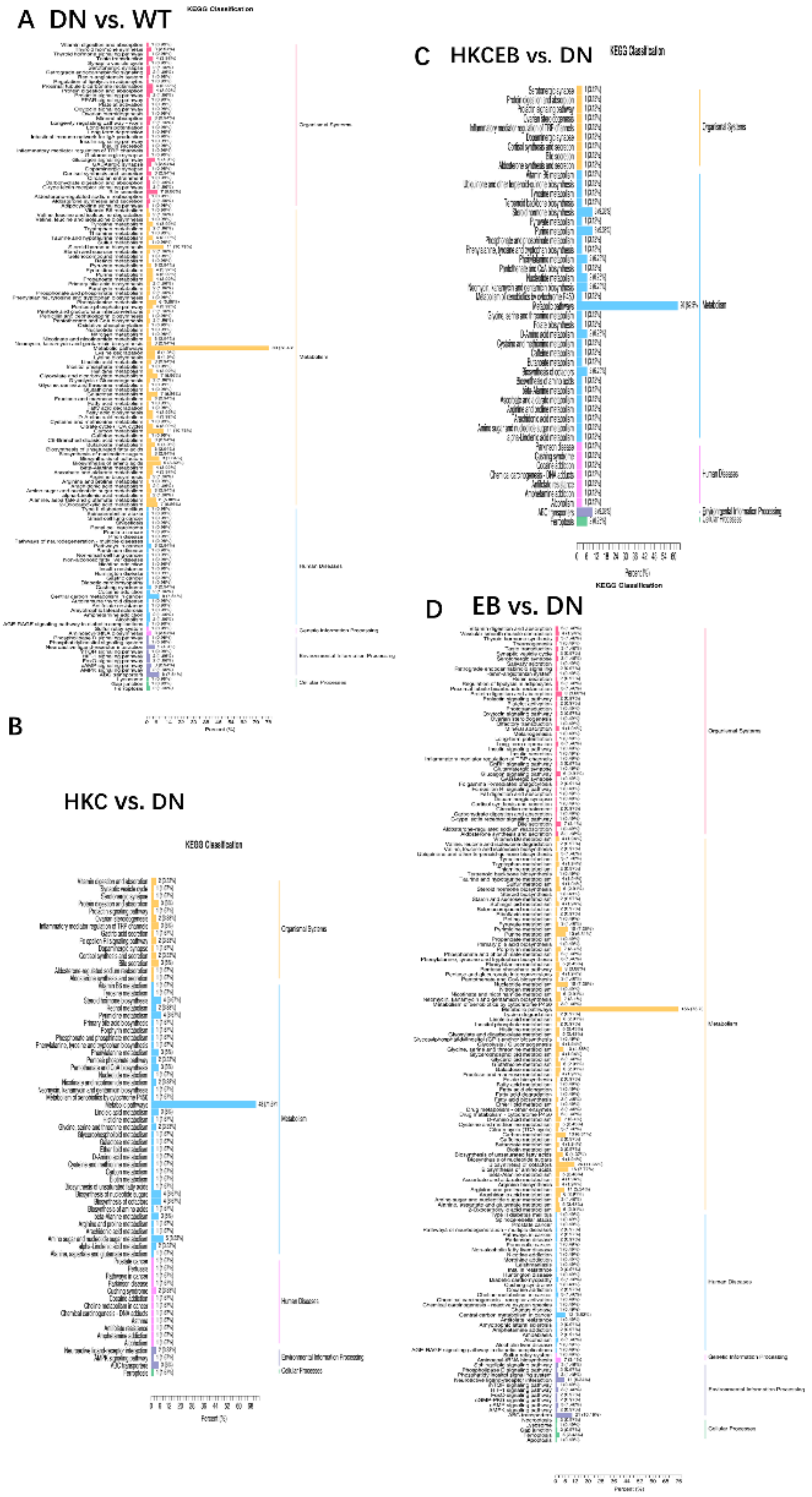

DN: diabetic nephropathy; WT: non-diabetic control; HKC: Huangkui capsule of *A. manihot*; EB: irbesartan; HKCEB: HKC combined with EB.

Fig. S6 The Venn diagram of the genes differentially expressed in kidneys

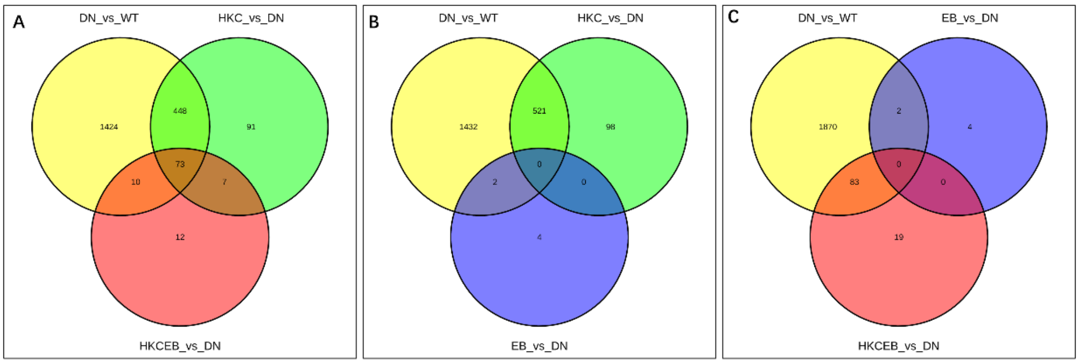

A total of 1955 expressed differentially genes were detected in kidneys of DN group compared to WT. Of them, 619 genes were founded in HKC, while only 6 in EB group. In HKCEB group, 102 genes were found. DN: diabetic nephropathy; WT: non-diabetic control; HKC: Huangkui capsule of *A. manihot*; EB: irbesartan; HKCEB: HKC combined with EB.

Fig. S7 TOP genes expressed differentially in kidneys of the db/db mice in HKC and HKCEB groups

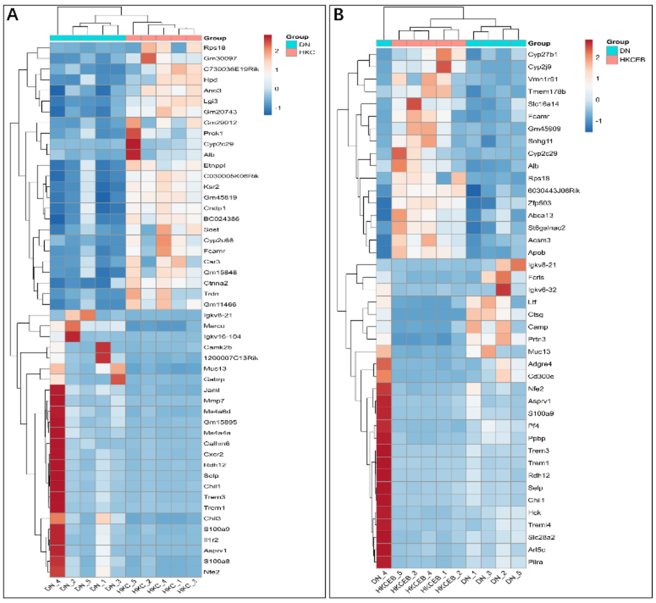

DN: diabetic nephropathy; HKC: Huangkui capsule of *A. manihot*; HKCEB: HKC combined with irbesartan.

Fig. S8 Volcanic map of the genes expressed differentially in kidneys

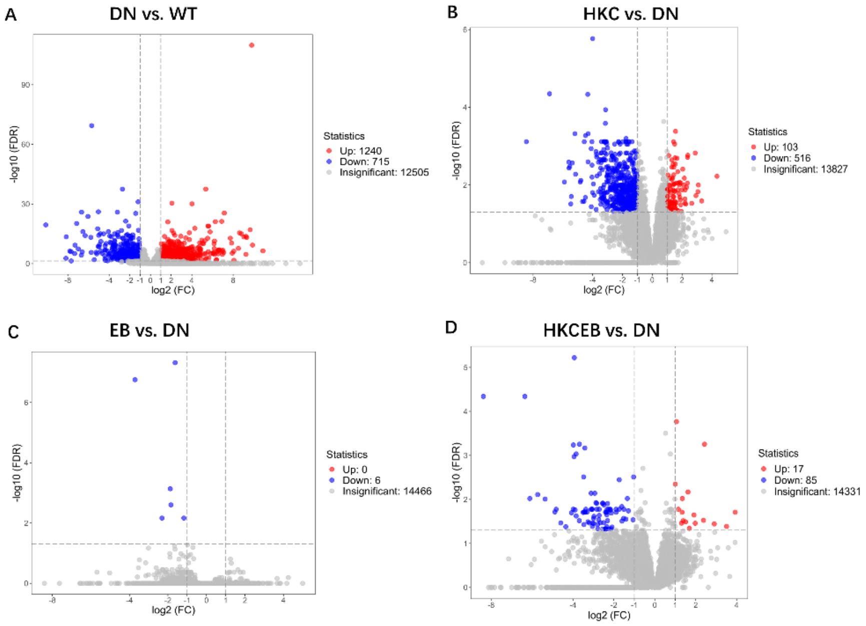

DN: diabetic nephropathy; WT: non-diabetic control; HKC: Huangkui capsule of *A. manihot*; EB: irbesartan; HKCEB: HKC combined with EB.

Fig. S9 GO enrichment analysis of differentially expressed genes in kidneys

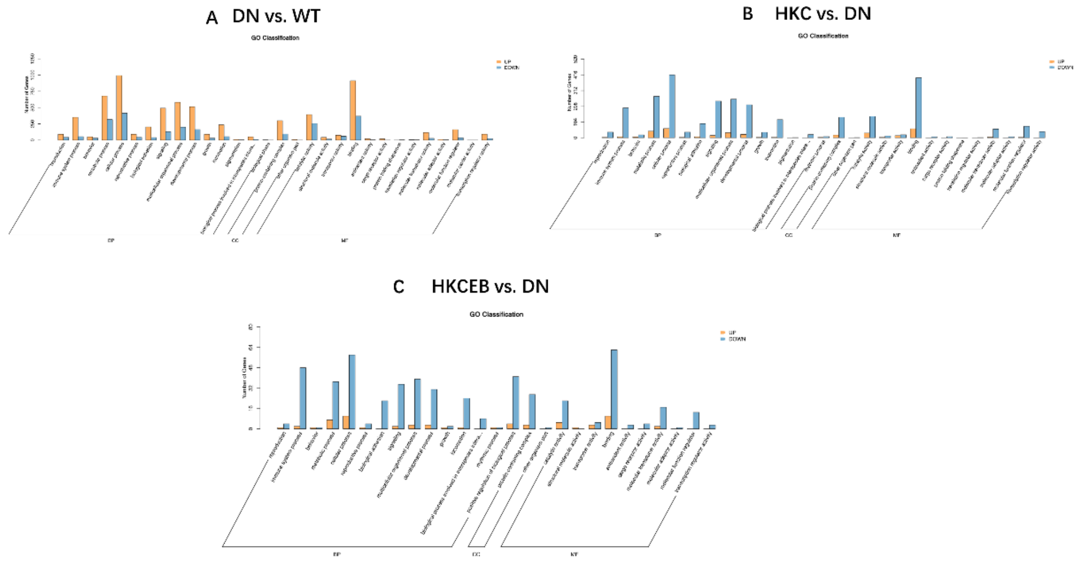

DN: diabetic nephropathy; WT: non-diabetic control; HKC: Huangkui capsule of *A. manihot*; EB: irbesartan; HKCEB: HKC combined with EB.

Fig. S10 KEGG pathways predicted based upon the genes expressed differentially in kidneys

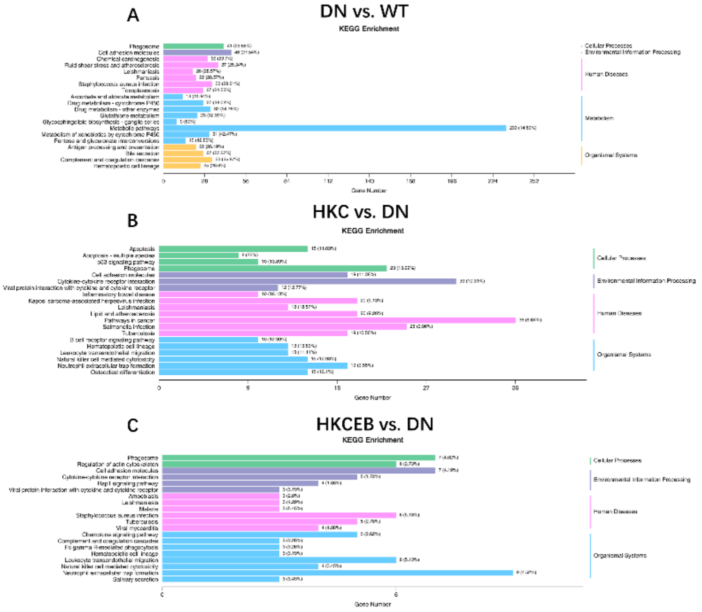

DN: diabetic nephropathy; WT: non-diabetic control; HKC: Huangkui capsule of *A. manihot*; EB: irbesartan; HKCEB: HKC combined with EB.

Fig. S11 KEGG pathways predicted based upon the genes expressed differentially in kidneys

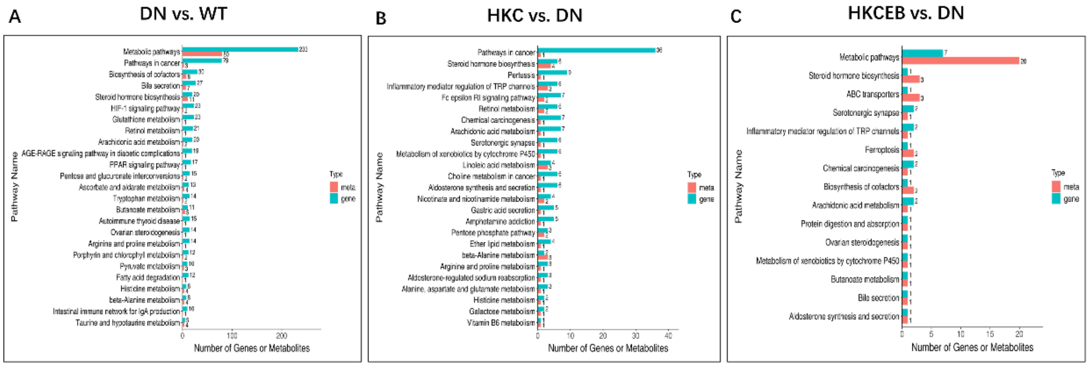

DN: diabetic nephropathy; WT: non-diabetic control; HKC: Huangkui capsule of *A. manihot*; HKCEB: HKC combined with irbesartan.

Fig. S12 The correlation between the metabolites in serum and the genes in kidneys

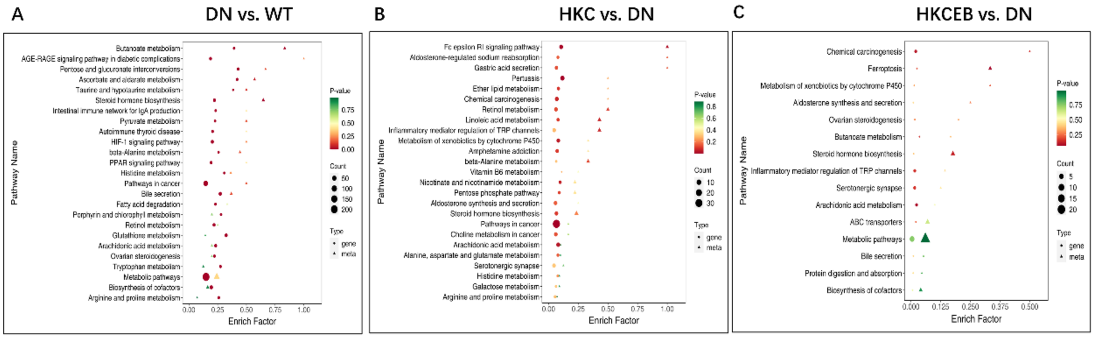

DN: diabetic nephropathy; WT: non-diabetic control; HKC: Huangkui capsule of *A. manihot*; HKCEB: HKC combined with irbesartan.
